# Changes in the transcriptional profile of transporters in the intestine along the anterior-posterior and crypt-villus axes

**DOI:** 10.1186/1471-2164-6-69

**Published:** 2005-05-10

**Authors:** Pascale Anderle, Thierry Sengstag, David M Mutch, Martin Rumbo, Viviane Praz, Robert Mansourian, Mauro Delorenzi, Gary Williamson, Matthew-Alan Roberts

**Affiliations:** 1 ISREC, Swiss Institute for Experimental Cancer Research, 1066 Epalinges s/Lausanne, Switzerland; 2 Swiss Institute for Experimental Cancer Research (ISREC) and Swiss Institute of Bioinformatics (SIB), NCCR Molecular Oncology, CH-1066 Epalinges s/Lausanne, Switzerland; 3 Nestle Research Center, Vers-chez-les-Blanc, 1000 Lausanne 26, Switzerland; 4 ISREC and Swiss Institute of Bioinformatics, 1066 Epalinges s/Lausanne, Switzerland; 5 Nestle Purina Pet Care, St. Louis, Missouri 63164, USA

## Abstract

**Background:**

The purpose of this work was to characterize the expression of drug and nutrient carriers along the anterior-posterior and crypt-villus axes of the intestinal epithelium and to study the validity of utilizing whole gut tissue rather than purified epithelial cells to examine regional variations in gene expression.

**Results:**

We have characterized the mRNA expression profiles of 76 % of all currently known transporters along the anterior-posterior axis of the gut. This is the first study to describe the expression profiles of the majority of all known transporters in the intestine. The expression profiles of transporters, as defined according to the Gene Ontology consortium, were measured in whole tissue of the murine duodenum, jejunum, ileum and colon using high-density microarrays. For nine transporters (Abca1, Abcc1, Abcc3, Abcg8, Slc10a2, Slc28a2, Slc2a1, Slc34a2 and Slc5a8), the mRNA profiles were further measured by RT-PCR in laser micro-dissected crypt and villus epithelial cells corresponding to the aforementioned intestinal regions. With respect to differentially regulated transporters, the colon had a distinct expression profile from small intestinal segments. The majority (59 % for p cutoff ≤ 0.05) of transporter mRNA levels were constant across the intestinal sections studied. For the transporter subclass "carrier activity", which contains the majority of known carriers for biologically active compounds, a significant change (p ≤ 0.05) along the anterior-posterior axis was observed.

**Conclusion:**

All nine transporters examined in laser-dissected material demonstrated good replication of the region-specific profiles revealed by microarray. Furthermore, we suggest that the distribution characteristics of Slc5a8 along the intestinal tract render it a suitable candidate carrier for monocarboxylate drugs in the posterior portion of the intestine. Our findings also predict that there is a significant difference in the absorption of carrier-mediated compounds in the different intestinal segments. The most pronounced differences can be expected between the adjoining segments ileum and colon, but the differences between the other adjoining segments are not negligible. Finally, for the examined genes, profiles measured in whole intestinal tissue extracts are representative of epithelial cell-only gene expression.

## Background

The absorption of biologically active compounds occurs via passive transcellular, paracellular and carrier-mediated transport mechanisms [1]. Analysis of the human genome sequence suggested the presence of 406 genes encoding ion channels and 883 genes encoding transporters [2]. Generally, these proteins establish the electrochemical gradient across membranes and provide the means for transporting electrolytes, amino acids, dipeptides, monosaccharides, monocarboxylic acids, organic cations, phosphates, nucleosides, and water-soluble vitamins [3,4]. Frequently, transporters play a direct role in the absorption of bioactive compounds from the intestinal lumen. The bioavailability of some compounds can depend significantly on carrier-mediated systems and, thus, are sensitive to drug-drug and drug-food interactions; however, these interactions tend to be more relevant for bioactive molecules with low bioavailabilities [5].

The mRNA expression profiles of several functionally-defined transporter families have already been measured by real-time PCR [6,7]; yet, for a majority of biologically active compounds it remains unknown which transporters play a role in their absorption. Although the human genome project has made the identification of most, if not all, genes encoding transporters and ion channels possible, only a few studies have focused on intestinal transporter expression using such genome-wide strategies [8-11]. Therefore, insight into the expression profiles of transporters along the intestinal tract enables a physiologically relevant assessment of their potential as drug- and nutrient-carriers.

Even less is known about the expression of transporters along the crypt-villus axis [12]. The extensive automated literature data mining by Olsen et al. [12] revealed that more transporters are known to be expressed in the villi than in the crypts. Many transporters that are villus-specific have been implicated in absorption processes, such as the oligopeptide transporter Slc15a1, facilitated glucose transporter Slc2a10, and the sodium/glucose cotransporter Slc5a1 [13]; whereas the crypt-specific, basolaterally expressed Na+/HCO3 co-transporter (Slc4a4) is essential for intestinal anion secretion. Similarly, the mulitrdrug resistance protein 1 (Abcc1), is crypt-specific, basolaterally expressed and acts as a secretion pump for various compounds [14,15]. On the other hand, P-glycoprotein (Abcb1) is villus-specific, expressed on the apical site and acts as well as a secretion pump for a variety of drugs [5,16]. Although exceptions may be found, one can assume that villus-specific transporters might be more efficient as mediators of absorption as their surface availability is more extensive.

Messenger RNA levels may not always correlate with the expression of encoded proteins [17]. Although use of proteomic techniques increasingly serves to resolve discrepancies between mRNA and protein levels, proteomics of integral membrane proteins still remains a challenge [18]. Also, protein levels do not necessarily correlate with protein activity. Various studies indicate that genomic profiling in combination with data mining of chemotoxicity databases can be an efficient strategy to identify new putative drug carriers [19,20].

A first step in identifying genes relevant to drug absorption in the intestine is to obtain a molecular catalogue of all expressed mRNAs. In this study, we used the high-density oligomer microarray by Affymetrix to measure the mRNA expression levels of genes expressed in four intestinal regions (duodenum, jejunum, ileum, and colon) in the mouse. We identified all genes with transporter activity according to the Gene Ontology (GO) consortium system that are represented on the microarrays. Then, we examined specific transporter classes that are significantly changed along the intestine and compared our findings to publicly available human microarray data. Finally, the mRNA expression of some selected transporters was measured along the crypt-villus axis using laser capture microdissection.

## Results

### "Carriers" and in particular "symporters" and "antiporters" are significantly changed along the A-P axis. However, he transporter class as a whole is not

Even though 41 % of all genes on the microarray that belong to the GO class "transporter activity" were differentially expressed along the intestine (modified ANOVA, p ≤ 0.05), the class itself, however, was not found significantly changed according to a Fisher's exact test (p ≤ 0.05).

**Figure 1:**
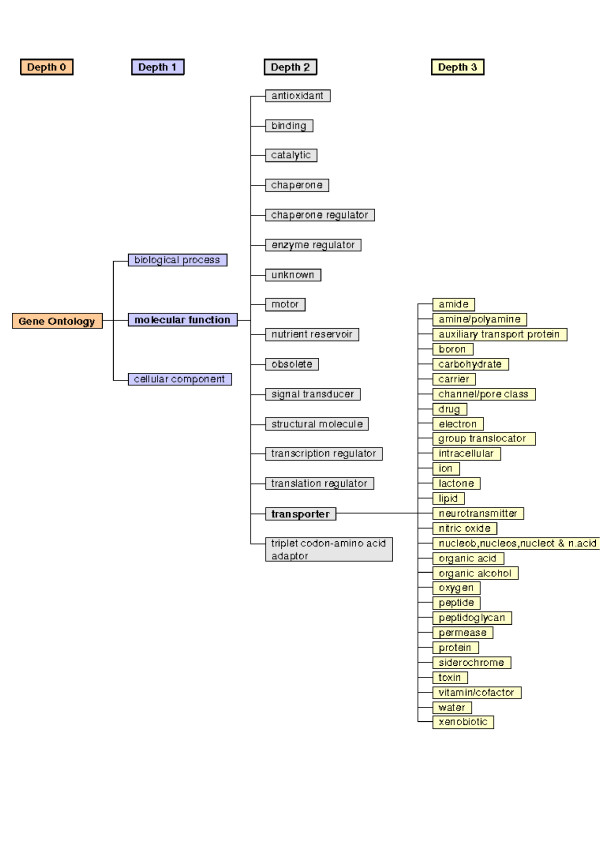
Selected branches of the classification system according to the Gene Ontology (GO) system.

In 8 of the 17 transporter classes represented on the microarrays (at depth 3 of the "transporter activity" mother class, cf. Figure 1) more than 50 % of the genes were differentially expressed along the intestine. But only the classes "carriers", "electron" and "protein" were significantly altered (cf. Table 1) with "carriers" being the only class whose subclasses showed significant changes. In particular, genes with "electrochemical potential-driven transporter activity", and more specifically "antiporters" and "symporters" were affected along the intestine. Although the class "channel/pore" was not significantly affected, it was the only transporter class (depth 3) besides "carriers" that contained subclasses that varied significantly, namely "chloride channel" (depth 7, 9 genes out of 13 were changed) and its subclass "voltage-gated chloride channel" (depth 8, 7 genes out of 9 were changed).

**Table 1:** Classification according to the Gene Ontology system of genes differentially regulated along the intestine.

**GO description**^a^	**Total (1-/G-F-Flag)**^b^	**UGs on Chips**^c^	**DJ**^b,d^	**JI**^b,d^	**IC**^b,d^	**SI C**^b,d^
molecular function	2717 (1772/760/185)	6890	659	897	897	2055
transporter activity	350 (244/75/31)	853	95	115	126	263
amine/polyamine	18 (11/2/5)	35	8	7	11	14
auxiliary transport protein	2 (2/0/0)	2	0	0	1	1
carbohydrate	8 (5/1/2)	18	2	4	7	8
carrier activity	124 (91/10/24)	262	35	41	55	98
electrochemical potential-driven transporter	57 (43/9/5)	104	20	27	35	47
porter	56 (42/9/5)	103	20	26	35	47
antiporter	11 (10/0/1)	16	4	6	9	11
cation\:cation antiporter	5 (5/0/0)	6	2	2	2	5
symporter	30 (18/7/5)	51	10	12	16	26
solute\:cation symporter	19 (10/5/4)	31	5	8	9	17
solute\:Na symporter	17 (8/5/4)	26	4	7	8	14
channel/pore class	64 (40/5/19)	211	15	19	22	47
electron	36 (272/7)	65	10	12	16	29
ion transporter	68 (49/5/14)	151	20	18	25	55
Lipid	6 (3/2/1)	16	2	3	5	4
neurotransmitter	8 (4/2/2)	12	2	3	5	7
nb/ns./nt/nucleic acid	2 (2/0/0)	4	1	0	1	1
Organic acid	22 (13/2/7)	43	8	9	11	16
Oxygen	2 (2/0/0)	5	0	1	0	0
Peptide	3 (3/0/0)	5	1	2	2	2
permease	1 (1/0/0)	5	0	0	0	2
Protein	78 (51/9/18)	163	18	17	13	56
Vitamin/cofactor	3 (1/2/0)	7	1	2	2	2
Water	1 (0/0/1)	2	0	0	1	1

^a^Transporter activity classes (depth 3) for which no changes were observed are not listed (number of UniGene clusters represented on the microarrays): amide (0), boron (0), drug (1), group translocator (0), lactone (0), nitric oxide (0), organic alcohol (0), peptidoglycan (0), siderochrome (0), toxin (0) and xenobiotic (0). ^b^Listed is the number of differentially regulated genes along the whole intestinal tract and more specifically between adjoining intestinal segments (p ≤ 0.05). Grey cells indicate significant changes in the GO nodes (p ≤ 0.05). ^c^Total number of UniGene clusters being represented on the three Mu74v2 GeneChips. ^d^Sum of all differentially expressed UniGene clusters, i.e., from type (1), (F) and (G).

### Expression profiles of transporters change along the whole intestinal tract, but most significantly between the ileum and colon

The number of significantly (p ≤ 0.05) regulated genes was not different when comparing the jejunum and ileum and the ileum and colon (cf. Table 1). However, comparisons of transporters revealed a trend that along the A-P axis the differences between the adjoining segments increased. Overall, this trend could also be observed in the transporter subclasses except from the "amine/polyamine", "ion transporter" and "protein" classes. For instance, the biggest subclass in absolute numbers of differentially changed transporters was the class "carrier". Along the intestine the difference between the adjoining segments increases clearly from 35 genes (duodenum vs. jejunum) to 41 (jejunum vs. ileum) to 55 (ileum vs. colon). The changes, however, are only significant (p ≤ 0.05) for the comparisons duodenum vs. jejunum and ileum vs. colon. Interestingly, no significant change was observed for the nodes of the jejunum vs. ileum comparison indicating that relative to all genes being differently expressed between the jejunum and the ileum the number of differently regulated transporters was not significantly higher.

Like the mother class "carriers", the number of differentially regulated "symporters" and "antiporters" also increased along the intestine. All changes were significant for both types of porters except for "symporters" in the comparison duodenum vs. jejunum (cf. Table 1).

### A similar number of transporters are over-expressed in the small and in the large intestine, but generally transporters are expressed at higher levels in the small intestine than in the colon

**Figure 2:**
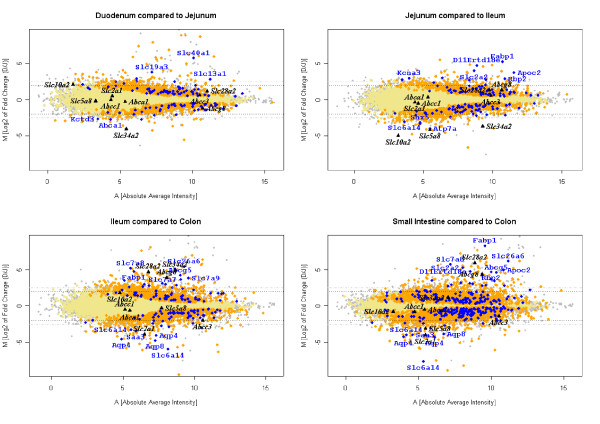
**A-D: M (log_2 _of fold change) vs. A (log_2 _of absolute average intensity) plots of the pair-wise comparisons between intestinal segments**. All genes are plotted in orange, and transporters for which both a significant difference was measured between the two segments of interest and had a "high" quality annotation are depicted in blue (p ≤ 0.05). In black are the transporters highlighted which were measured in the crypts and the villi. Grey lines indicate thresholds for fold changes of four fold (M = ± 2) or five fold (M = ± 2.5), respectively.

In order to identify transporters which could be interesting candidate carriers for bioactive compounds in the various segments, we examined expression profiles along the intestinal tract. Comparing the expression levels of transporter genes or genes involved in transporter activity in the small intestine and the colon revealed that similar numbers of genes were more highly expressed in either the small or the large intestine. However, small intestinal transporters were clearly more over-expressed (cf. Figure 2). The majority of differentially expressed transporters are members of the solute carrier super family (Tables 2 and 3). The most pronounced change (fold changes > 300), however, was observed for fatty acid binding protein 1 (Fabp1). Among the transporters being more than 20 times over-expressed in the small intestine were the neurotransmitter transporter Slc6a19, the sulfate transporter pendrin-like protein 1 (Slc26a6), L-type amino acid transporter 2 (Slc7a8), facilitated glucose transporter 2 (Slc2a2), the apolipoprotein C-II (Apoc2), the D11Ertd18e, the retinol binding protein 2 (Rbp2), sterolin 1 and 2 (Abcg5 and Abcg8) (genes are listed in decreasing order of the fold change). The Y(+)L-type amino acid transporter 1 (Slc7a7), the concentrative nucleoside transporter co-transporter (Slc28a3), the B(0,+)-type amino acid transporter 1 (Slc7a9) were more than 10 times over-expressed. In the colon, the most over-expressed transporter was the neurotransmitter transporter Slc6a14 which was more than 200 times higher expressed in the colon compared to the small intestine. Aquaporin 4 (Aqp4), serum amyloid A 3 (Saa3), the neurotransmitter transporter Slc6a14, the facilitated glucose transporter 1 (Slc2a1) and aquaporin 8 (Aqp8) were more than 10 times over-expressed. The concentrative nucleoside transporter 2 (Slc28a2, Cnt2) was the top five most over-expressed transporter in the small intestine compared to the colon, but its annotation quality was only classified as "medium".

**Table 2:** Differently regulated (p ≤ 0.05) solute carriers with a minimum absolute fold change of four in pair-wise comparisons of adjoining intestinal segments. Bold cases indicate fold changes of five or higher (Means ± SDs, log2 scale, n = 3 or 9, respectively.).

**Gene Name**	**D > J**	**D < J**	**J > I**	**J < I**	**I > C**	**I < C**	**SI > C**	**SI < C**	**Literature**^a^	**Gene aliases**	**References**
Slc10a2				5.0 ± 1.2					Mouse and Human: Highly expressed in I	ASBT; ISBT	[36, 37, 53]
Slc12a4					2.6 ± 0.5		2.9 ± 0.5		-	KCC1; RBCKCC1	
Slc13a1	2.8 ± 0.4			**2.2 ± 0.5**					Mouse: D/J ~ C < I; Human: only in kidney	Nas1; NaSi-1	[54, 55]
Slc14a1					**2.2 ± 1.2**		**2.1 ± 1.2**		Human: Expressed in C; Rat: expressed in C	UT-B	[56, 57]
Slc17a6		**2.2 ± 0.4**							Rat: abundant in I	DNPI, VGLUT2	[58]
Slc17a7	**2.2 ± 0.**4								-	Vglut1	
Slc19a3	3.8 ± 0.9								Human: D > J > C > I	ThTr2	[59]
Slc1a1				2.4 ± 0.2	2.8 ± 0.2				Mouse: SI; rat: high in distal SI; human: intestine	EAAC1, EAAC2, EAAT3, MEAAC1	[60-62]
Slc20a1						2.8 ± 0.4			Mouse: Present in D	Glvr-1, Glvr1	[63]
Slc25a22					2.8 ± 0.3		2.7 ± 0.3		Human: SI	-	[64]
Slc26a2								2.5 ± 0.3	Human: C > SI	Dtd, ST-OB	[65]
Slc26a6					5.4 ± 0.2		6.1 ± 0.3		Mouse: D, J, I > C; Human: D>C>J~I, SI > C	CFEX; Pat1	[66, 67]
Slc28a3					3.5 ± 1.2		3.5 ± 1.1		Human: D > J > I > C	Cnt3	[68]
Slc2a1						3.9 ± 0.6		3.10 ± 0.3	Rat: Low expressed in SI; Human: expressed in colon carcinoma	Glut1; Glut-1	[69, 70]
Slc2a2			4.0 ± 0.4		2.6 ± 0.5		5.2 ± 0.8		Human: expressed in SI	Glut2; Glut-2	[71]
Slc2a5			2.6 ± 0.4				3.2 ± 0.7		Mouse: expressed in SI; Human: expressed in J	Glut5; Slc5a	[72, 73]
Slc34a2		4.0 ± 0.3		3.7 ± 0.2	4.9 ± 0.6				Mouse: expressed in SI and colon; Human: SI specific	Npt2b; NaPi-2b	[35, 74]
Slc35a1						**2.1 ± 0.2**		2.7 ± 0.3	-	-	
Slc39a10				**2.2 ± 0.3**					-	-	
Slc40a1	5.7 ± 0.3					2.5 ± 0.5			Mouse:high in D, not detected in I; Rat: expressed in D, C; Human: highly expressed in D	MTP; Ol5; Pcm; Dusg; Fpn1; MTP1; IREG1; Slc11a3; Slc39a1	[75-77]
Slc4a7	2.4 ± 0.2					2.5 ± 0.2			Human: expressed in SI and C	NBC3; NBCn1	[78]
Slc5a1					**2.1 ± 0.1**		2.7 ± 0.2		Mouse and Human: Specific for SI; Rat: possible expression in C	Sglt1	[79, 80]
Slc6a14				3.0 ± 1.9		6.0 ± 1.8		7.7 ± 0.7	Mouse: SI < C; Human: weak in C, absent in SI	ATB0plus; CATB0plus	[81, 82]
Slc6a19					6.7 ± 0.6		6.8 ± 0.6		-	B<0>AT1	
Slc6a4					3.1 ± 0.1		3.1 ± 0.2		Mouse and human: Intestinal enterocytes	Htt; Sert; 5-HTT	[83]
Slc7a7					3.6 ± 0.1		3.7 ± 0.2		Mouse: expressed in J, I; human: highly expressed in SI	my+lat1	[84, 85]
Slc7a8		**2.1 ± 0.4**			5.2 ± 0.7		5.7 ± 0.8		Mouse: expressed in SI	LAT2	[86]
Slc7a9					3.8 ± 0.2		3.4 ± 0.2		Mouse and human: expressed in SI	CSNU3	[85]
Slco1b2	**2.1 ± 1.1**								-	OATP2; Oatp4; lst-1; OATP-C; mlst-1; Slc21a6; Slc21a10	

^a^D = Duodenum, J = Jejunum, I = Ileum, C = Colon, SI = Small intestine, - = No data found in the literature

**Table 3:** Differently regulated (p ≤ 0.05) non-SLC transporters with a minimum absolute fold change of four in any pair-wise comparison of adjoining intestinal segments. Bold cases indicate fold changes of five or higher (Means ± SDs, log2 scale, n = 3 or 9, respectively.).

**Gene Name**	**D>J**	**D<J**	**J>I**	**J<I**	**I>C**	**I<C**	**SI>C**	**SI<C**	**Literature**^a^	**Gene aliases**	**References**
Aldh9a1					2.4 ± 0.1				-	ESTM40; TMABA-DH	-
Apoc2			3.7 ± 0.5		2.5 ± 0.5		4.9 ± 0.6		Mouse: expressed in intestine; Rabbit: expressed in J, D; Human: expressed in J	-	[87-89]
Aqp4						4.6 ± 0.8		4.3 ± 0.3	Mouse: present in SI in crypt cells, and C in epithelial surface cells; rat: SI, C	MIWC; mMIWC	[90, 91]
Aqp5	2.9 ± 0.4								Rat: D only	-	[91]
Aqp8						4.8 ± 0.6		3.9 ± 0.4	Rat: SI and C	-	[91]
Atp2a3								**2.1 ± 0.2**	Human: C > SI	Serca3; SERCA3b	[92]
Atp7a	2.5 ± 0.5			4.0 ± 0.4					Human: Highly expressed in D	Mo; br; Blo; I14; blotchy; mottled; MNK; brindled	[93]
Chrna1	2.5 ± 0.4								-	Acra; Achr-1	-
Chrne					**2.0 ± 0.4**				-	Acre	-
Csng					**2.0 ± 0.8**				-	Csn1s2a; Csn1s2b	-
D11Ertd18e			4.7 ± 0.5				4.7 ± 1.0		-	-	-
Fabp1			5.2 ± 1.1		4.7 ± 1.1		8.3 ± 0.10		Mouse: D, proximal J>I, nothing in distal I; Human: highest in J, present in D, J, I, C	Fabpl; L-FABP	[94]
Gfpt1				**2.2 ± 0.4**				2.4 ± 0.4	Mouse: expressed in SI and C; Human: C > SI	GFA; GFAT; Gfpt; GFAT1	[95, 96]
Gfpt2							**2.1 ± 0.4**		Human: SI and C very low expressed	-	[96]
Gria1	**2.2 ± 0.5**								-	Glr1; Glr-1; GluRA; Glur1; HIPA1; GluR-A; Glur-1	-
Grm3						2.4 ± 1.2		**2.3 ± 1.2**	-	Gprc1c; mGluR3	-
Gsr				**2.1 ± 0.3**					-	Gr1; Gr-1	-
Kcna3			2.9 ± 0.6					**2.0 ± 0.4**	-	Mk-3; Kv1.3; Kca1-3	-
Kcnk6									Mouse: C > SI	Toss; Twik2;	[97]
Kctd3		2.7 ± 1.2	2.7 ± 1.3						-	NY-REN-45	-
Pkd2								2.4 ± 0.5	-	-	-
Pln								2.6 ± 0.5	-	PLB	-
Rbp2			2.8 ± 0.2		**3.0 ± 0.3**		4.7 ± 0.5		-	Rbp-2; Crbp-2; CrbpII	-
Rbp7					2.9 ± 0.3		2.7 ± 0.3		-	CRBP-III	-
Saa3						3.5 ± 0.6		4.0 ± 0.4	Mouse and Human: Expressed in SI and C	Saa-3	[98]
Stard5						2.9 ± 1.0			-	D7Ertd152e	-
Stx1b1					**3.0 ± 1.2**				-	Stx1bl	-
Trpm7				**2.0 ± 1.0**					-	CHAK; PLIK CHAK1; Ltpr7; Ltrpc7;	-
Xdh							2.8 ± 0.3		Mouse: Strong in SI	Xor; Xox1; Xox-1	[99]

^a^D = Duodenum, J = Jejunum, I = Ileum, C = Colon, SI = Small intestine, - = No data found in the literature

### There are particular transporters that are clearly segment-specific. However, most differentially regulated transporters are similarly expressed along the whole small intestine

A more detailed pair-wise comparison between the adjoining segments indicated that the differences between adjoining segments, regarding changes of expression levels of transporters, increased along the intestine (cf. Figure 2, Tables 2 and 3). Most transporters that were differently expressed between the small intestine and the colon were similarly expressed along the length of the small intestine, but differently changed between the ileum and the colon; however, some transporters were clearly expressed in a region-specific manner. Ferroportin 1 (Slc40a1) was strongly over-expressed in the duodenum compared to the other segments. Besides Slc40a1, the intestinal phosphate transporter (Slc34a2) had the most pronounced fold change between jejunum and the duodenum. The expression level of Slc34a2 increased along the small intestine, but was again more lowly expressed in the colon than in the ileum. Fabp1 is similarly expressed in the duodenum and the jejunum, but significantly (p ≤ 0.05) decreased in the ileum and even more in the colon. Besides the Na+ dependent ileal bile acid transporter (Slc10a2) and Slc34a2, the sodium transporter Slc5a8, a tumor suppressor gene [21], was also more highly expressed in the ileum compared to the jejunum, but similarly expressed in the colon. On the other hand, Fabp1 is the most highly expressed gene involved in transport activity in the jejunum compared to the ileum. This is followed by D11Ertd18e, which has been associated to the sugar transporter super family based on its protein structure, and the facilitated glucose transporter member 2 (Slc2a2). Tables 2 and 3 show all transporters that were differently regulated along the intestine and whose fold changes were at least four-fold. Members of the ABC transporter family have not been integrated in the tables as they have been presented earlier in detail [10].

### Most transporters are similarly expressed in mice and humans

In order to assess if the expression levels in the mouse are a suitable estimator for the situation in humans our data were compared to publicly available human microarray data obtained with a custom-array. Overall, 20 % of all annotated transporters in mouse were compared. Previous studies have shown that there is a good correlation between data obtained with the Affymetrix platform and the data obtained with this custom-array [22].

The majority of common orthologous transporters were similarly expressed in the small intestine and the colon in both mice and humans. Some genes, however, were no less than four-fold up-regulated in at least one segment in the mouse, but were not identified as being differently regulated in humans except for low affinity Na-dependent glucose transporter Slc5a2 (cf. Table 4). Other studies in humans not using microarrays, however, suggest that most of these genes have a similar expression profile in mice and humans (Table 4) indicating that the fold changes measured with the custom-array may to a certain extent underestimate the true fold changes as shown earlier [22].

**Table 4:** Comparison of the relative expression levels of transporters in mice and humans. Only genes with at least one four-fold difference in pair-wise comparisons are shown

**Gene Name**	**Human**^a^		**Mouse**^a^		
	**SI > C**	**SI > C**	**D > C**	**J > C**	**I > C**

AQP5			**x**		
SLC19A3			**x**		
SLC26A6		**x**	**x**	**x**	**x**
SLC28A3		**x**	**x**	**x**	**x**
SLC2A2		**x**	**x**	**x**	
SLC34A2					**x**
SLC37A4				**x**	
SLC6A4		**x**		**x**	**x**
SLC7A7		**x**		**x**	**x**
SLC7A8		**x**	**x**	**x**	**x**
SLC7A9			**x**	**x**	**x**

	**C > SI**	**SI < C**	**D < C**	**J < C**	**I < C**

AQP4		**x**	**x**	**x**	**x**
AQP8		**x**	**x**	**x**	**x**
PKD2				**x**	
SLC26A2^b^			**x**		
SLC10A2				**x**	
SLC2A1		**x**	**x**	**x**	**x**
SLC6A14			**x**		**x**
SLC35A1				**x**	
SLC40A1				**x**	
SLC5A1^c^	**x**				

^a^Published expression data are listed in tables 2 and 3. D = Duodenum, J = Jejunum, I = Ileum, C = Colon, SI = Small intestine, - = No data found in the literature. ^b^According to Haila et al. [65] highly expressed in the human colon and low in the human small intestine. ^c^According to Wright et al. [79] specific for human and mouse small intestine.

### The regulation of expression in whole intestinal tissues is a good indicator of the combined changes in epithelial crypt and villus cells

In order to assess whether the expression profiles of genes in whole tissue reflect their expression in the epithelium, we determined the expression profile of 12 genes in villus and crypt cells using a combined laser micro-dissection and RT-PCR approach. To validate this method we measured a marker for the villi (aminopeptidase N/Anpept), for crypts in the small intestine, i.e., Paneth cells (defensin related cryptdin 5/Defcr5) and crypts in the small intestine and colon (caudal type homeo box 1/Cdx1).

**Figure 3:**
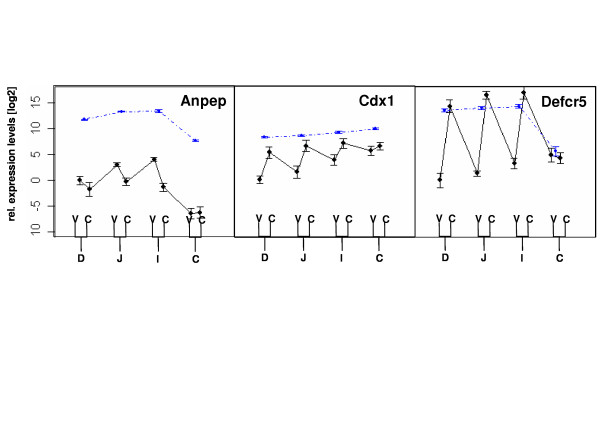
**Expression profiles of tissue-specific markers along the intestine**. Blue, dash-dotted lines indicate the relative expression in the four intestinal segments (D, J, I, C) using microarrays (mean ± SD, n = 3 pools of 10 mice each). Black, continuous lines indicate the relative expression in the four intestinal segments in the crypts and villi using RT-PCR (mean ± SD, n = 5). Please note that connecting the black lines between crypts and villi are not logical connections and only for visual support.

Based on the expression levels of the three crypt/villus markers, RNA obtained from laser dissection of the small intestine seems to be highly enriched of region specific material, i.e. villi samples do not contain a notable portion of RNA originating from crypt cells (cf. Figure 3).

In order to assess the expression profiles of transporters measured in whole intestinal tissue with microarrays, we verified the mRNA levels of nine transporters (Abca1, Abcc1, Abcc3, Abcg8, Slc10a2, Slc28a2, Slc2a1, Slc34a2 and Slc5a8) measured in the same RNA sample preparation by RT-PCR. The expression profiles measured with microarray and RT-PCR had a good concordance. In general, we observed that the profiles measured in the whole tissue in the four segments reflected the average expression in the crypts and the villi.

### Influence of the crypt-villus axis on the expression of transporters

**Figure 4:**
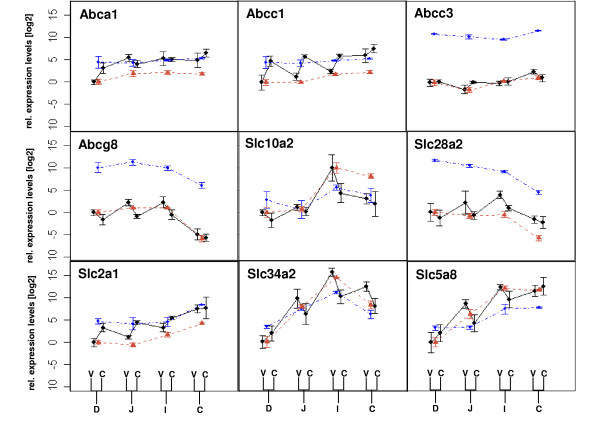
**Expression profile of selected transporters along the intestine**. Blue, dash-dotted lines indicate the relative expression in the four intestinal segments (D, J, I, C) using microarrays and brown dashed lines using RT-PCR (mean ± SD, n = 3 pools of 10 mice each). The microarray results for the ABC family members are according to Mutch et al. [10]. Black, continuous lines indicate the relative expression in the villi (V) and crypts (C) as determined with laser dissected material and RT-PCR (mean ± SD, n = 5 individual mice). The black lines between crypts and villi are meant only for visual support.

To test if there is a relationship between the expression profile along the intestine and along the crypt-villus axis, we measured the expression of a subset of transporters in laser-dissected material. We selected transporters which were, based on microarray and RT-PCR results of the whole tissue (cf. Table 5), not differentially expressed along the intestine (Abca1, Abcc1, Abcc3), small intestine specific (Abcg8), large intestine specific (Slc2a1), either specific for the anterior small intestine (Slc28a2) or posterior part of the whole intestine (Slc10a2, Slc34a2, Slc5a8). All nine transporters examined in laser-dissected material tended to conserve their tissue specificity along the intestine (cf. Table 5, Figure 4). In other words, none of the examined transporters was crypt-specific in one segment and villus-specific in another. Abcc1 and Slc2a1 were crypt-specific, whereas Slc34a2 villus-specific in at least three segments. Abcg8 and Slc28a2 showed a villus-specificity in the posterior part of the small intestine, while Slc5a8 was only in the jejunum villus-specific and Slc10a2 in the ileum.

**Table 5:** Segment and tissue specificity of transporters along the A-P and crypt-villus axis.

**Transporter**	**Segment-Specificity**^a,M^	**Segment-Specificity**^a,R^	Tissue-Specificity^b^			
			**Duodenum**	**Jejunum**	**Ileum**	**Colon**

Abca1	D=J=I=C	J=I=C>D	Crypts>Villi	ND	ND	ND
Abcc1	D=J=I=C	I=C>D=J	Crypts>Villi	Crypts>Villi	Crypts>Villi	ND
Abcc3	D=C>J=C>I=C	I=C>D=J	ND	Crypts>Villi	ND	Crypts>Villi
Abcg8	J>I=D>C	D=J=I>C	ND	Villi>Crypts	Villi>Crypts	ND
Slc10a2	I>C>D=J	I>C>D=J	ND	ND	Villi>Crypts	ND
Slc28a2	D>J>I>C	D=J=I>C	ND	Villi>Crypts^x^	Villi>Crypts^x^	ND
Slc2a1	C>D=J=I	C>I>D=J	ND	Crypts>Villi	Crypts>Villi	Crypts>Villi^x^
Slc34a2	I>C>J>D	I>C=J>D	ND	Villi>Crypts	Villi>Crypts	Villi>Crypts
Slc5a8	C>I>D=J	C=I>J>D	ND	Villi>Crypts	ND	ND

^a^Expression in the four intestinal segments (D, J, I, C) was measured in whole tissue using microarrays (M) and RT-PCR (R) (n = 3, p ≤ 0.05). ^b^Tissue specificity was determined in laser dissected crypts and villi (n = 5, where p ≤ 0.05 or if indicated with a superscript "*" p ≤ 0.10). "ND" indicates that no significant difference in expression levels were measured between the crypt and villus epithelial cells.

## Discussion

A crucial step in classifying genes into different molecular functions is the use of a consistent and universal classification system and a precise annotation of Affymetrix probe sets. Therefore, we included the following features in our analysis: i) Mapping of single probes to all transcripts referred to in the UniGene database and attributing of annotation tags to each probe set, and ii) assigning of flags to differentially regulated probe sets. Similar to Chalifa-Caspi et al. [23], we have seen that the annotation provided by Affymetrix (NetAffx) is not entirely accurate. In the Affymetrix annotation a probe set is represented by at most one UniGene cluster, while, conducting our own mapping of Affymetrix probes onto UniGene, we observed that in a number of cases multiple UniGene identifiers can be associated to a given probe set.

Based on our quality criteria, 48 % of all probe sets in the murine genome were found to have a "high" quality annotation on the microarrays used in this study. Thus, selecting only "high" quality genes establishes a high degree of confidence regarding the correct annotation of probe sets.

We observed that the genes on the microarrays are not represented by an equal number of probe sets. Hence, by counting each probe set as a unit in the GO classification system the way it is done by a majority of GO classification programs such as MappFinder, OntoExpress and the one provided by NetAffx, a bias may be introduced [24,25]. Moreover, to our knowledge, none of the programs publicly available provides a confidence assessment regarding the significantly regulated functional classes. Therefore, we developed a software, the so-called ISREC Ontologizer (Io), that takes these factors into account. The use of the flags (i.e., 1, F, G) provided with the Io software permits such an assessment to be made. As indicated in Figure 2, a significant portion of the results have a flag "F" and were, therefore, considered ambiguous. Some of these "flagged" results, based on UniGene identifiers, could be due to the fact that some of the probe sets are associated with the same UniGene cluster, but are specific for different splice variants. However, we observed a similar portion of ambiguous results (i.e., flag "F") when doing the classification based on RefSeq identifiers and concluded that the ambiguity associated with flagged genes may stem from probe design rather than the differential expression of splice variants [26].

Some transporters are subject to post-translational modification and of course, one of the processing steps is translocation to the membrane. However, if one considers mRNA to be representative of function, then the majority of transporters can be expected to have similar activity along the length of the intestine. In this regard, it is interesting to note that a remarkable fraction of transporters annotated with "carrier activity" are differentially regulated along the A-P axis and could therefore significantly influence the absorption of carrier-mediated bioactive compounds. Our GO classification results indicate that, in the context of the whole transcriptome, transporters do not seem to be an especially dynamically regulated class, which differs slightly to the conclusions made by Bates and colleagues [9]. However, a direct comparison is difficult to make, as the classification system used by Bates et al. was not in accordance with the official GO classification system, and this may underlie the different biological conclusions made between the two studies. Furthermore, more importantly, while that study included only 4 % of all annotated transporters (6), this study covers 76 % and, provides the first extensive overview of the genomic profiles of transporters in the intestine. Similar to their study, the most pronounced difference between adjoining regions was observed for the ileum and the colon. Although, in general, the differentially expressed transporters were more highly expressed in the small intestine than in the colon, a surprisingly high number of transporters were identified as being more highly expressed in the colon than in the small intestine [13]. Many of these transporters have been described as highly expressed in the colon and involved in the bi-directional transport of electrolytes and fluids, which is the principal role of the colon [27]. However, the majority of known drug and nutrient transporters or carriers of bioactive compounds [28] are not differently expressed between the small intestine and the colon.

The exploitation of transporters, which are highly expressed in the colon or at similarly high levels as in the small intestine is especially interesting for compounds such as proteins and peptides, which are susceptible to the high enzymatic activity present in the small intestine [29]. For example, Slc6a14, which transports neutral and amino acids, might be an interesting candidate to be targeted for carrier-mediated absorption. Generally, we observed that most of the differentially regulated "carriers" were not specifically over-expressed in any of the three small intestinal segments, but overall more highly expressed in the small intestine versus the colon. Thus, the ileum, which contains smaller concentrations of pancreatic enzymes, could be a suitable tissue for compounds that are sensitive to the activity of pancreatic enzymes.

Genes classified as having "carrier", "channel", "ion transport" and "electron" and "neurotransmitter" activity are most likely to have different expression levels between the small intestine and colon and may, hence, play a more significant role in the difference of active drug and nutrient transport in these two anatomically distinct regions.

The expression profiles of the most strongly regulated genes (i.e. absolute fold change of 4 between the segments) agreed with previous findings in mice (cf. Table 2). Moreover, comparing transporter expression profiles in mice and humans suggests that gut transcriptional profiles obtained in mice are a valid estimator for the situation in humans (Table 2 and Table 3).

There are several methods that can be used to characterize the patterns of expression of a target gene. Traditionally, labeled antisense specific probes have been widely used for *in situ *hybridization to detect specific gene expression in the intestinal epithelium [12,30]. This technique, however, is very labor-intensive when several candidates are being tested. There are several techniques used to isolate the epithelial layer from the lamina propria of the intestine [31]. These methods use mechanical procedures and detergents and/or EDTA to detach the epithelial layer. Separation of crypts from villi epithelium can be achieved following adequate separation procedures. However, alterations of gene expression patterns depending on the procedure as well as cross-contamination from different tissue compartments are possible. Similarly, laser dissection microscopy has been developed to isolate single cells [32]. A key step in this procedure is the selection of an adequate fixation procedure that prevents RNA degradation and preserves tissue histological integrity [33,34]. The protocols used in the present study allows the assessment of a minimally altered gene expression pattern that is concordant with *in situ *hybridization experiments (M. Rumbo unpublished data).

Within this context, our data suggest that the changes in expression profiles measured in whole intestinal tissue extracts are a suitable predictor for changes in epithelial cell-only gene expression along the crypts and the villi; however, it should be mentioned that all genes in our comparison were already known to be expressed in the epithelium. This good correlation could reflect the fact that these genes are solely expressed in the epithelium or that the epithelium contributes much more to the isolated RNA than underlying layers of intestinal tissues and, therefore dominates the genomic profile of the intestine. Thus, for genes which are not solely expressed in the epithelium the prediction based on measurements in whole tissue might not be appropriate.

For some compounds, depending on the enzymatic stability and solubilization characteristics, targeting of carriers in distinct intestinal regions may selectively improve their absorption and bioavailability. Thus, we analyzed the expression profiles of known drug carriers along the intestine. The major part of inorganic phosphorus (P_i_) absorption from the small intestine occurs via a Na^+^-dependent phosphate co-transporter, NaP_i_-II_b _encoded by the gene SLC34A2. *In vivo *pharmacokinetic studies in rats have indicated that foscarnet is transported across the enterocytes by the NaP_i_-II_b _system and thus acts as a competitive inhibitor of P_i _uptake [28]. Slc34a2 mRNA has been shown to be expressed in the small intestine and the colon [35], which is in accordance with our findings. Our studies further demonstrate that the expression significantly increases along the A-P axis of the small intestine. As a consequence, the most efficient transport rate is expected to arise in the ileum. In addition to the findings by Hilfiker et al. [35] that Slc34a2 protein is expressed on the apical membrane of mature enterocytes in the duodenum, we observed that Slc34a2 mRNA is villus-specific along the whole intestine except for the duodenum, reinforcing the concept that transporters expressed in the villi epithelium tend to be implicated more in absorptive processes.

Slc10a2 (ASBT), the primary carrier for Na^+^-dependent bile salt uptake from the intestinal lumen by the ileum, has been shown to be present in the brush-border membrane of the terminal part of the ileum, but little is known concerning its regulation along the intestine [36,37]. Various studies indicate that based on substrate examples ASBT could be an important target for increasing the bioavailability of various bile acid conjugates [28]. Our findings confirm that Slc10a2 is especially highly expressed in the ileum compared to proximal small intestinal regions; however a comparable level was measured in the colon. Interestingly, only in the ileum was Slc10a2 identified as being villus-specific which could be an indication for its specific role in the ileum.

The four members of the ABC families, Abcg8, Abca1, Abcc1 and Abcc3, that we studied are known to act as secretion pumps in the intestine [14,38,39]. In accordance with earlier findings, Abcc3 expression increases from the anterior to posterior part of the intestine [10,40]. On the other hand, our findings for Abcc3 did not indicate an increase in mRNA expression from the crypts to the villi as shown by Rost et al. [40] in rats. The mRNA levels of Abcc1 along the crypt-villus axis were in accordance with protein expression [14]. Generally, all four members may play a significant role along the whole small intestine as secretion pumps, whereas in the large intestine, Abcg8 is not expected to be involved in the secretion of compounds.

The transporter Slc28a2 is known to be involved in the transport of nucleoside analogues such as some antiviral compounds [41]. Nucleosides are relatively hydrophilic molecules and their ability to be transported across cell membranes is a critical determinant of their metabolism [42]. Our data indicate that similar uptake rates can be expected along the whole intestine when targeting this carrier, as it is similarly expressed in the gut. On the other hand, Slc2a1, which was significantly (p ≤ 0.05) more highly expressed in the colon, might be an appropriate drug-carrier for compounds sensitive to pancreatic enzymatic degradation. It has to be noted though that the protein product of SLC2A1, GLUT1, is not detectable in healthy human colon tissue, but only in colon cancer serving as a marker for poor prognosis [43,44]. Thus, it may be an interesting drug-target/drug carrier in individuals with colon cancers.

In contrast to GLUT1, SLC5A8 has been shown to be highly expressed in the colon and to act as a tumor suppressor gene. SLC5A8 is silenced by methylation in most human colon tumors and reintroduction of this gene leads to growth suppression [21]. Recent studies have shown that SLC5A8 acts as a Na+-coupled transporter for short chain fatty acids and monocarboxylates. Its transport rate is strongly inhibited by various drugs such as ibuprofen. We have shown for the first time that this gene is expressed at similar levels in the distal regions of the gut and seems to be preferentially expressed in the villi of the small intestinal enterocytes. Summarizing, this Na+-coupled transporter may be involved in the active transport of known monocarboxylate drugs and serve as a valuable drug-carrier for new drugs along the whole intestine.

## Conclusion

In conclusion, we have characterized the mRNA expression profiles of 76 % of all currently known transporters along the A-P axis of the gut. We have identified various transporters that could serve as carriers for biologically active compounds. The significant regional specificities are likely to correspond to functional differences along the length of the intestinal tract.

Finally, we have presented a comprehensive analysis of regional variations in gene expression using whole gut tissue that is sufficiently sensitive to provide a good assessment of relative changes in regional gene expression in epithelial cells; however, for the functionality of a given transporter to be unequivocally determined, it will be critical to examine these epithelial cells in a cell-type specific manner.

## Methods

### Animals and tissue handling

8 week-old male Hsd:ICR(CD-1) mice (Harlan, Netherlands) were provided by AMS Biotechnology (Lugano, Switzerland). Thirty animals were divided into 3 pools. The small intestine was extracted and divided into three sections, where the first 2–3 cm after the stomach comprised the duodenum, the middle third the jejunum, and the section before the ileo-ceco-colic junction comprised the ileum. The colon was treated as a single intestinal section.

### Gene expression analysis using the murine Mu74v2 GeneChips

Gene expression was measured as previously described [10]. Briefly, total RNA extracts were provided by AMS Biotechnology (Lugano, Switzerland) and RNA extraction was performed identically for each pool of mice. RNA was then re-purified, according to manufacturer's instructions using the Nucleospin kit and contaminating genomic DNA was removed by DNase1 treatment (Macherey-Nagel AG, Oensingen, Switzerland). The quality and quantity of RNA was determined using Agilent 2100 Bioanalyser (Agilent Biotechnologies, Germany) (1.6 – 2.0 ratio for 28/18S and no significant amount of metabolized products). For each murine gut tissue section, 5 μg total RNA was used as the starting material for all individual samples. Labeling and fragmentation of cRNA, array hybridization and scanning was performed according to the protocol by Affymetrix. Fluorescence values from scanning were analyzed with Affymetrix Gene Expression Analysis Software (MAS 5.0). The complete data set is publicly available at  through the accession number GSE849.

### Laser dissection microscopy

The mRNA expression levels of selected transporters (Abca1, Abcc1, Abcc3, Abcg8, Slc10a2, Slc28a2, Slc2a1, Slc34a2 and Slc5a8) were measured in the crypts and villi of the intestinal mucosa of five 8 week-old male Hsd:ICR(CD-1) mice (Harlan, Netherlands). To assess the performance of the micro-dissection, specific markers for the crypt-villus axis were used (Anpept for villi, Cdx1 for crypts and Defcr5 for crypts in the small intestine, namely for Paneth cells) [45-48]. Immediately after sacrificing the animals, the intestinal tract was removed and regions classified similar to the microarray tissue sample definitions (cf. above). Sections of one cm length were cut and incubated overnight in zinc fixative/sucrose 30% solution (5g ZnCl_2_, 6g ZnAc_2_X2H_2_O, 0.1g CaAc_2 _in 1L of 0.1M Tris pH 7.4). Afterwards, they were embedded in OCT and frozen by immersion in liquid nitrogen. 20 μm frozen sections were cut and mounted on Leica membranes for dissecting microscopy (Leica Microsystems, Wezlar, Germany), fixed in 96 % ethanol for 30 s and colored for an equivalent time with Mayer's hematoxylin solution. Membranes were then rinsed in water for 1 minute, transferred for 10 s to 70 % ethanol, followed by 96 % ethanol and air dried. Samples were processed using a laser dissecting microscope (Leica Microsystems), coupled to a CCD camera. Microdissected samples were collected in a tube cap placed below the sample holder. Samples were collected in 20 μL RNA lysis buffer. Total RNA was extracted using the total RNA extraction Nucleospin II kit by Machery-Nagel (Oensingen, Switzerland).

### Real-time PCR (RT-PCR)

Reverse transcription (RT) was performed using Superscript II (Gibco BRL). RT-PCR amplification was performed using an ABI 5700 machine (Applied Biosystems, Foster City, CA, USA) with the following thermal cycling conditions: 2 min at 50°C, 10 min at 95°C, followed by 40 cycles of 95°C for 15 s and 60°C for 1 min. The quality of all RNA samples were examined with the Agilent 2100 Bioanalyser (Agilent Biotechnologies, Germany). All samples were standardized to equal RNA concentration using the RiboGreen RNA quantification kit (Molecular Probes, Leiden, Netherlands) and measured in duplicates. Cycle to cycle fluorescence emission was monitored and quantified using the GenAmp software provided by Applied Biosystem. Data was normalized to Gapdh. Primer probes were purchased from Applied Biosystems as Assays-on-Demand^SM ^(Applied Biosystems, Foster City, CA, USA)

### Data analysis

#### GEA analysis and ANOVA of microarray data

Differential gene selection was determined using a Global Error Assessment (GEA) method of analysis [49]. Genes were considered as differentially regulated along the intestinal tract if the p-value = 0.05. GEA assumes that the variance of the absolute intensity of a given gene is a function of the absolute intensity. Therefore, rather than treating each gene on the microarray as a unique and unrelated element, neighboring genes are binned into groups of 200 based on similar intensity signals and the mean squared error is calculated for each bin. For tables 2 and 3 standard deviations were calculated for each gene independently.

#### Analysis of RT-PCR data

A one-way ANOVA and pair-wise comparisons based on Tukey's Honest Significant Difference method (p- values as indicated in the figures and tables) were used to confirm differences in gene expression.

### Annotation of probe sets and functional classification of genes

Whereas in NetAffx the UniGene identifier is determined according to the probe set's representative sequence (i.e., the sequence used to design the probe set), we have independently mapped the probes and attributed a quality tag depending on the level of specificity [50]. In order to better assess the significance of the findings it is necessary to know if the different probe sets supposed to represent the same transcript give the same result and if all the probes in a probe set still represent one and the same transcript according to the most recent reconstruction of the mouse transcriptome. Therefore all probes of the Affymetrix Mu74v2 GeneChip were mapped to the latest release of UniGene clusters (Unigene build number 137, EST db, HTC db, RefSeq db and mRNA db) by an in-house developed tag-matching software as described earlier [51].

On this basis, every probe set was assigned to one of four probe set "quality classes". The top class "high", the only one used in this work, requires that all the probes of the same probe set have perfect sequence matches to at maximum two UniGene clusters, which was the case for 17625 probe sets, as defined elsewhere [51].

### Structure of classification program

Following the UniGene annotation, probe sets were associated to the nodes they represent in the gene function tree defined by the Gene Ontology (GO) project (, version of April 2004). For probe sets mapping to two different UniGene clusters, both clusters were considered for GO annotation.

A Java program called ISREC ontologizer (Io) was used to efficiently browse and analyze the data. Io is a general purpose Java program for classification of microarray results according to an annotation system , which will be described in detail elsewhere. In short, for a given list of (differentially regulated) probe sets (features), Io shows their distribution over all GO classes subdivided by classes of probe set quality and evaluates the statistical significance of overrepresentation of a GO class. This analysis can be done with the features as individual entities or pooling in groups those that represent the same UniGene cluster to study the degree of agreement between probe sets of the same cluster. In this case, three counts are provided for each GO class: 1/F/G ("one"/"flagged"/"good"). The "One" counter is incremented when the group to which a feature belongs contains only that feature. For groups with more than one feature, the "good" counter is incremented when all features are present in the selected list of probe sets; otherwise the "flagged" counter is incremented and indicates an ambiguous experimental result.

Significant changes (p ≤ 0.05) for each GO node were calculated using a Fisher's exact test using the "1" or "F" or "G" counts [52].

### Comparison to human data

The mouse microarray data were compared to publicly available microarray data (the data set is available at  through the accession number GSE1368) of the human small intestine and the colon. For all "high quality" Affymetrix probe sets the corresponding human homologue genes were identified on the human chip using the LocusLink identifier and the Homologue mapping table . 224 orthologous transporters were identified as being in common between the two platforms. Then, the relative fold changes observed in the mouse were compared to the human data. In case of multiple probes representing the same gene average expression values were used. In the case of the mouse data, relative fold changes were calculated by comparing the expression levels of each independent small intestinal segment to the colon and the average of all three segments to the colon as it was unclear which small intestinal segment was compared to the colon in the human study. Only genes four times or higher expressed in any segment compared to another segment were considered in this comparison.

## Authors' contributions

PA contributed to the design and coordination of the study, carried out the laser dissection, comparison of mouse and human microarray data and expression data in the literature, participated in the development of the ISREC ontologizer, was responsible for high level analysis of microarray data and drafted the manuscript. TS participated in the development of the ISREC ontologizer and the annotation of the probe sets, and implemented its methods in the Java environment. DMM contributed to the design of the study and participated in the editing of the manuscript. MR set up the protocols for the LDM and participated in the laser dissecting. VP was mainly responsible for the annotation of the probe sets. RM performed the global error assessment (GEA). MD participated in the development of the ISREC ontologizer and participated in the editing of the manuscript with respect to the statistical methods. GW contributed to the design and planning of the study, to the discussion and interpretation of results, and to the writing of the manuscript. MR contributed to the original conception, design and coordination of the study. Further contribution was made to choosingproject goals, experimental approaches, developing technical resources towards advancement of the project, and finally revising the manuscript prior to publication.

## Note

In the following, the term "transporter" will refer to all genes that are classified as genes with "transporter activity" according to the GO system. At the date of analysis the ATP-binding cassette (ABC) transporters were not classified as genes with "transporter activity". Thus, this class has not been integrated the GO analysis. However, in the analysis of expression levels on a gene by gene basis the ABC transporters have been integrated.

Please note that according to the official human HUGO and mouse MGI system we will refer to human gene symbols in upper case, and to mouse genes with a first letter in capital and the subsequent ones in lower case.
